# Respiratory entrainment related reverse triggering in mechanically ventilated children

**DOI:** 10.1186/s12931-024-02749-7

**Published:** 2024-03-25

**Authors:** Robert G.T. Blokpoel, Ruben B.R. Brandsema, Alette A. Koopman, Jefta van Dijk, Martin C.J. Kneyber

**Affiliations:** 1 Department of Paediatrics, Division of Paediatric Intensive Care, Beatrix Children’s Hospital, University Medical Center Groningen, P.O. Box 30.001 9700 RB, Groningen, CA 62 the Netherlands; 2 Critical Care, Anesthesia, Peri-operative medicine & Emergency Medicine (CAPE), University of Groningen, Groningen, the Netherlands

**Keywords:** Reverse triggering, Neuromechanical coupling, Double triggering, Phase-locking, Pediatrics

## Abstract

**Background:**

The underlying pathophysiological pathways how reverse triggering is being caused are not fully understood. Respiratory entrainment may be one of these mechanisms, but both terms are used interchangeably. We sought to characterize reverse triggering and the relationship with respiratory entrainment among mechanically ventilated children with and without acute lung injury.

**Methods:**

We performed a secondary phyiology analysis of two previously published data sets of invasively mechanically ventilated children < 18 years with and without lung injury mechanically ventilated in a continuous or intermittent mandatory ventilation mode. Ventilator waveforms, electrical activity of the diaphragm measured with surface electromyography and oesophageal tracings were analyzed for entrained and non-entrained reverse triggered breaths.

**Results:**

In total 102 measurements (3110 min) from 67 patients (median age 4.9 [1.8 ; 19,1] months) were analyzed. Entrained RT was identified in 12 (12%) and non-entrained RT in 39 (38%) recordings. Breathing variability for entrained RT breaths was lower compared to non-entrained RT breaths. We did not observe breath stacking during entrained RT. Double triggering often occurred during non-entrained RT and led to an increased tidal volume. Patients with respiratory entrainment related RT had a shorter duration of MV and length of PICU stay.

**Conclusions:**

Reverse triggering is not one entity but a clinical spectrum with different mechanisms and consequences.

**Trial registration:**

Not applicable.

## Background

Reverse triggering (RT), as a subtype of patient-ventilator asynchrony (PVA), is increasingly being reported in mechanically ventilated adults and children [1, 2]. A key feature of this type of asynchrony is that the patient effort is generated after the start of a mandatory breath. RT may lead to breath stacking and excessive pleural swings, thereby contributing to volutrauma, barotrauma and diaphragm dysfunction [3, 4]. While it is increasingly being identified, the effects of RT on clinical outcome have not been fully elucidated. This is in part due to the fact that clear definitions regarding different phenotypes of RT and when RT is truly harmful do not exist.

**Fig. 1:**
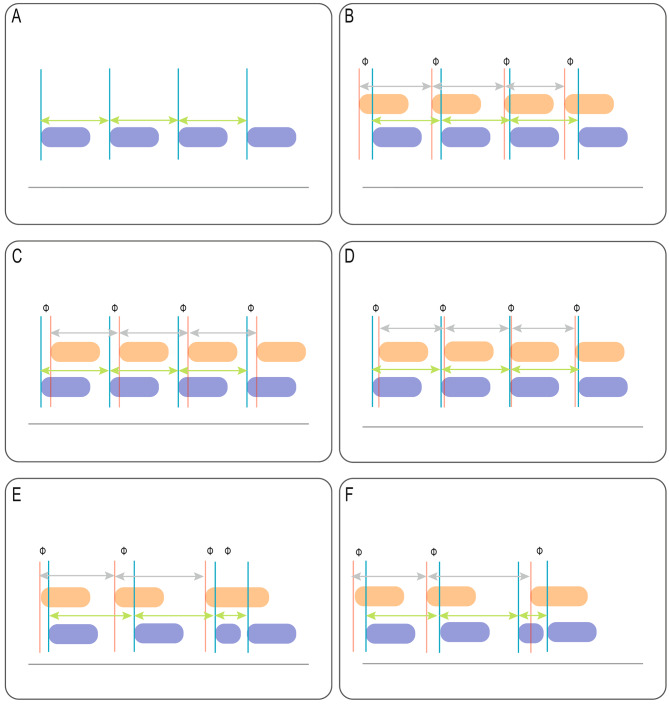
Graphical examples of type of breaths. Different graphical examples of type of breaths and its relationship with phase angle, breathing interval and coefficient of variation (CoV). CoV calculations are based upon multiple breathing cycles. Due to the schematic examples these cycles are not shown in the figure. Purple rectangle: ventilator pressurization, orange rectangle: patient effort, blue line: start of ventilator breath, red line: start of patient effort, gray arrow: patient breathing interval (TTOTNEU), green arrow: ventilator cycle (TTOTMECH), Φ: phase angle. **A**: Time triggered mandatory breath and no patient effort. Ventilator cycle (TTOTMECH) remains stable without variabilty. CoV calculations of the interval between mandatory breaths will be < 15%. Due to no patient efforts no phase angle calculations could be made. **B**: Spontaneous breathing pattern with changing timing in breathing interval (TTOTNEU) and different phase angles for each breath. Phase angles remain positive. CoV calculations for phase angle and breathing interval are showing no sign of respiratory entrainment and will be > 15%. **C**: Reverse triggering as a direct response to a time triggered breath. Both breathing interval (TTOTNEU) and phase angle are showing no variation. Phase angles will a be negative. CoV calculations of the breathing interval and phase angles are < 15%. **D**: Reverse triggering as part of respiratory entrainment. Patient triggered breaths and reverse triggering breaths are showing an alternating pattern. Phase angles will be positive, zero or negative. Breathing interval is showing little variabilty. CoV calculations for the breathing interval will be < 15% and for phase angle 10–15%. **E**: Double triggering during spontaneous breathing. Double triggering is patient triggered. CoV calculations of the breathing interval and phase angle are > 15%. **F**: Reverse triggering with double triggering during spontaneous breathing without respiratory entrainment. During double triggering the patient effort followed a time triggered mandatory breath. CoV calculations from both breathing interval and phase angle are > 15%. Hence, showing no relation with respiratory entrainment

The mechanism causing RT is not completely understood [5]. It has been proposed that respiratory entrainment may play an important role in the development of RT. Unfortunately, the terms RT and respiratory entrainment are frequently used interchangeably. However, it is important to differentiate between RT and respiratory entrainment, as there are different physiological pathways underlying RT and respiratory entrainment. Respiratory entrainment is a form of physiological neuromechanical coupling in which the subject’s breathing frequency matches an external stimulus (i.e., lung inflation) creating a fixed patient respiratory rhythm [6–8]. By definition, during respiratory entrainment patient efforts preceding the ventilator breath (i.e., spontaneous breathing pattern) and patient efforts after the ventilator breath (reverse triggering) occur alternately in this type of neuromechanical coupling [6]. This is in contrast to RT in which patient efforts are only seen after the ventilator breath and spontaneous breaths are not observed (Fig. 1). In addition, during respiratory entrainment, a patient breathing pattern shows less variability compared to the variation during a non-entrained spontaneous breathing pattern [6–9]. Thus, in case of RT caused by entrainment (i.e., entrained RT) both patient breaths (patient-triggered and reverse) should show little variance in breathing timing and the subjects neural breathing frequency should match the mandatory breath rate set on the ventilator (Fig. 1). It may be surmised that in these circumstances, as being a normal physiological response, RT may not result in injurious volume delivery.

**Fig. 2:**
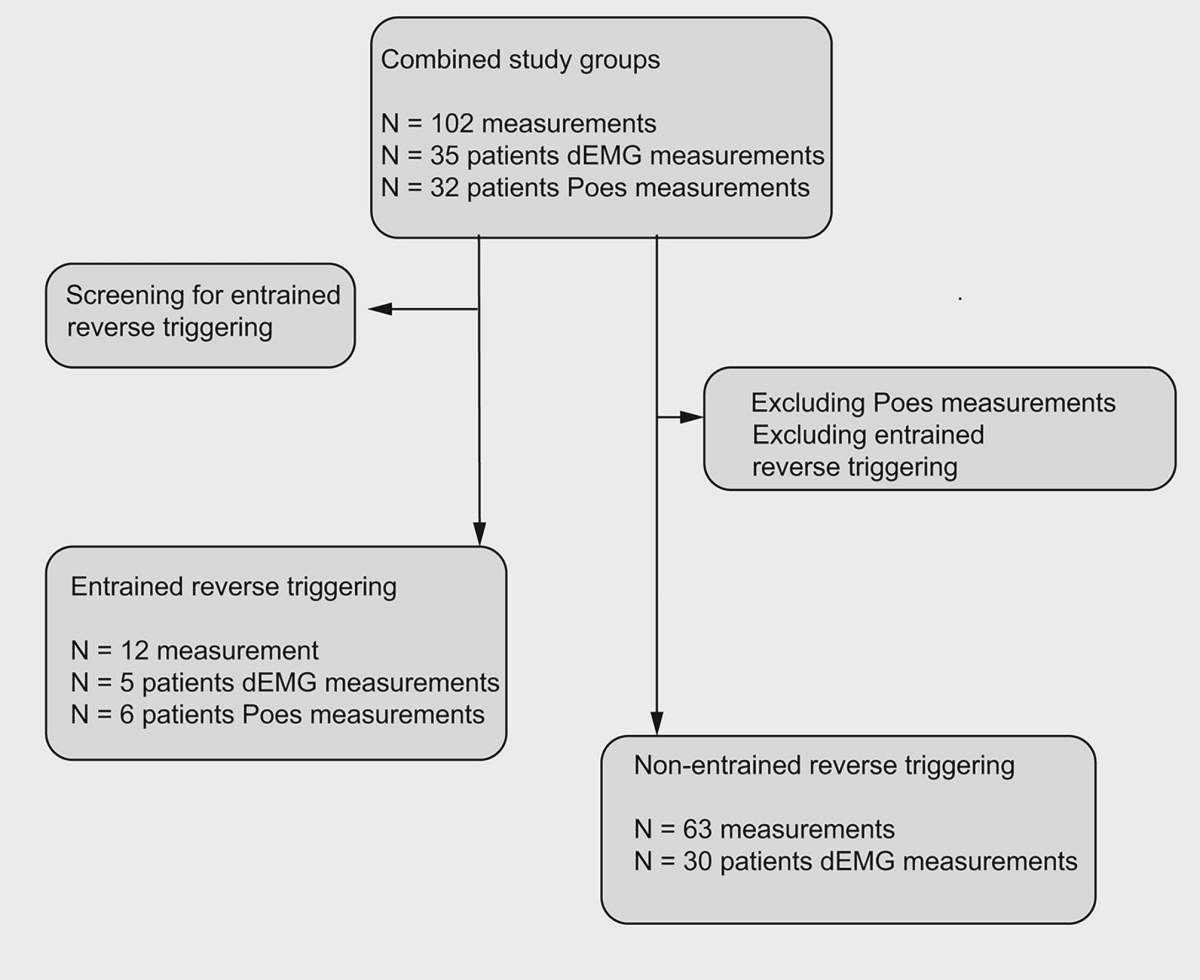
Study flow chart diagram. Study flow chart diagram entrained and non-entrained reverse triggering

At the same time, RT is not always caused by respiratory entrainment [10, 11]. These non-entrained RT subtypes have been classified according to timing (i.e., RT occurring during the inspiratory or expiratory phase of the breathing cycle) [12], presence or absence of breath stacking, or as a non-repetitive, single event (so-called non-entrained RT) (Fig. 2) [1, 13, 14]. Like entrained RT, the mechanisms causing non-entrained RT are not fully understood. Some authors describe this subtype of RT without entrainment by the ventilator as premature triggering or as complete desynchronization. Hence, it can be hypothesized that other factors like ventilator settings or delivered tidal volume (Vt) can induce non-entrained RT [15].

Because of different physiological pathways it could be of importance to discriminate between respiratory entrainment induced RT and non-entrained RT. We therefore sought to characterize RT and its relationship with respiratory entrainment and to characterize single non-entrained RT events in a heterogeneous cohort of mechanically ventilated children. For this purpose, by using surface electrical activity of the diaphragm and oesophageal pressure manometry, we identified differences between time triggered mandatory breaths, patient triggered breaths and RT breaths (entrained and non-entrained) (Table 1). We also aimed to identify risk factors for single non-entrained RT events.

**Table 1:** Definition of terms used

Term Definition	
Coefficient of variation	Statistical measure used to assess the relative variability of a dataset expressed as a percentage. CoV = (standard deviation/mean) * 100.
Double triggering	Two consecutive ventilator cycles separated by a short expiratory time, i.e., half of the inspiratory time or less.
Entrained reverse triggering	Breathing effort after the start of a ventilator cycle in a regular pattern (four consecutive reverse triggered breaths). Pattern occurring in a 1:1, 1:2 or in 1:3 ratio. During entrainment the patient is cycling between patient triggered and reverse triggered breaths. Breathing pattern is showing low variability (i.e. coefficient of variation < 15%).
Non-entrained reverse triggering	Breathing effort after the start of a time triggered mandatory breath occurring as a single event.
Patient-triggered breath	Patient effort (increase in electrical activity of the diaphragm/negative deflection in the oesophageal pressure tracing) followed by a ventilator pressurization.
Phase-angle	Calculation to describe the time duration or delay between a patient effort and the initiation of the ventilator pressurization. A phase angle of 0° means that the patient effort starts at the same time as the initiation of the ventilator pressurization. A phase angle of 180° means that the patient’s effort starts halfway between the start of two mandatory breaths. Phase angle = (start patient effort – start ventilator pressurization/breathing cycle) * 360°.
Respiratory entrainment	Form of neuromechanical coupling in which the subject’s breathing frequency matches an external stimulus creating a fixed patient respiratory rhythm with less variability (i.e. coefficient of variation < 15%).
Reverse triggering	Patient effort generated after a time triggered mandatory breath.
TTOT_MECH_	Duration of the ventilator breath. Time (sec) period between the start of a time cycled mandatory breath until the start of the consecutive time cycled mandatory breath
TTOT_NEU_	Duration of a patient triggered ventilator cycle. Time (sec) between the start of a patient effort (based on dEMG signal or oesophageal pressure) until the start of the consecutive patient effort
Time triggered mandatory breath	Ventilator pressurization without any signs of a patient effort.

## Methods

We used two data sets of invasively mechanically ventilated children with and without lung injury [16]. The need for consent for the first study was waived by the Institutional Review Board (IRB) of the University Medical Center Groningen [16]. In this study recordings were made of the ventilator flow-time, pressure-time and oesophageal pressure-time scalar. The second study was approved by the IRB (NL46097.042.13), and written informed consent was obtained from the parents or legal caretakers. For this study recordings were made of the ventilator flow-time, pressure-time and the electrical activity of the diaphragm. Anonymous data from both studies were aggregated for the analyses presented here.

Both data sets included prospectively collected 30-minutes recording (Jan – Nov 2018 and Feb – July 2015) from patients < 18 years [16]. Because during respiratory entrainment patient’s breathing frequency matches an external stimulus only patients able to trigger the ventilator were included. Patients with congenital or acquired neuromuscular disorders, severe traumatic brain injury (i.e., Glasgow Coma Score < 8), uncorrected congenital heart disorder, chronic lung disease and severe pulmonary hypertension were excluded. In addition for this secondary analysis data from subjects who were on a ventilation mode without a set mandatory ventilator breath rate (i.e., a continuous spontaneous ventilation [CSV] mode) were excluded.

The aggregate data included anonymized patient characteristics (age, gender, weight, admission diagnosis), ventilator settings (mode, pressure above PEEP (PAP), PEEP, mean airway pressure (Pmean), pressure support (PS), expiratory tidal volume (Vte ml/kg, actual bodyweight), set mandatory breath rate, inspiratory time and fraction of inspired oxygen (FiO_2_), and oesophageal pressure), and clinical characteristics; prior use of neuromuscular blockade (NMB) for moderate/severe acute respiratory distress syndrome (ARDS), amount of analgesia-sedation in the 4 h preceding the recording, Comfort B score as marker of patient comfort, endotracheal tube (ETT) size and percentage of ETT leakage [17, 18]. If ETT leakage exceeded 18% patients were excluded. All patients were ventilated with one type of ventilator (Avea, Vyaire Medical, Yorba Linda, USA). In patients < 15 kg a proximal flow sensor was used. Ventilator data were acquired through the Ventilator Open XML Protocol (VOXP) interface at a sampling rate of 100 Hz. The electrical activity of the diaphragm (EAdi) was measured through transcutaneous recording of the electromyographic diaphragm signal (dEMG) (and other respiratory muscles i.e., the intercostal and abdominal muscles) at a sampling rate of 500 Hz using the Porti (TMSi, Oldenzaal, The Netherlands) using one pair of Ag/AGCl electrodes (EasyTrode TM Pre gelled Electrodes, Multi Bio Sensors Inc, El Paso, USA) bilaterally placed at the costo-abdominal margin at the nipple line for the dEMG. Data acquisition and analysis was peformed using Polybench (Applied Biosignals GmbH, Weener, Germany) and Matlab R2018a (Mathworks, Natick, MA, USA).

### Definition of entrainment, neural breathing frequency, entrained and non-entrained reverse triggering

We applied previously published definitions of entrainment and RT [1, 6–8, 19]. Briefly, RT was defined as a patient effort after the start of a time triggered mandatory breath, either displaying a regular pattern (i.e., entrained RT) or as a single event (i.e. non-entrained RT) (Fig. 1D and F). Pattern of entrained RT could occur in a 1:1, 1:2 or in 1:3 ratio, thus one time triggered mandatory breath followed by a patient effort, one out every two time triggered mandatory breaths followed by one patient effort or one out every three time triggered breaths followed by one patient effort. We considered entrained RT if there were four or more consecutive RT breaths [19]. During respiratory entrainment the patient breathing frequency matches an external stimulus creating a fixed patient respiratory rhythm. Due the nature of this fixed rhythm, breathing variability, of both patient triggered and reverse triggered breaths, during respiratory entrainment will be lower compared to the breathing variability during normal spontaneous breathing [6–9]. To express the degree of entrainment (i.e. loss in breathing variability) the coefficient of variation calculation (CoV) is used. This statistical measure us used to assess the relative variability and is expressed as a percentage (standard deviation/mean*100). The time interval (sec) of the breathing cycle and CoV were calculated for time triggered mandatory breaths (TTOT_MECH_), patient triggered (TTOT_NEU_) and RT breaths [1]. CoV < 15% was considered as respiratory entrainment [9]. For each entrained RT breath, non-entrained RT breath, and patient triggered breath, we calculated phase angle and its CoV. Examples of phase angle, breathing, CoV for each type of breath and used definitions are described in Fig. 1; Table 1. To determine the characteristics of patient triggered and entrained RT breaths, we calculated for each single breath tidal volume (Vte), oesophageal pressure-time-product (PTP), delta oesophageal pressure (ΔPes), integrated EAdi signal (dEMG_INT_) and amplitude from the EAdi (ΔdEMG) signal. PTP was calculated by integrating the area under the oesophageal pressure versus timetracing form the beginning until the end of inspiration [20].

### Definition of reverse triggering with breath stacking

To detect RT with breath stacking, all double triggering events were manually annotated. Double triggering was defined as two consecutive ventilator cycles separated by a short expiratory time, i.e., half of the inspiratory time or less [21]. Each double triggering event was labeled as patient-triggered or as a ventilator initiated (Table 1).

### Data selection and analysis

First, we screened the full 30-minute recordings for stable RT events (Fig. 2). If detected up to five stable reverse triggering patterns per recording were randomly selected. To identify differences in breaths characteristics for each individual patient, if available, an equal number of patient triggered, entrained RT triggered and time triggered mandatory breaths were used for analytical purposes.

To study the occurrence of single non-entrained RT and neural mechanical coupling, we only analyzed data from patients with dEMG-recordings. Patients with entrained RT events were excluded. These patients were screened for non-entrained RT events. Patients with merely oesophageal pressure measurements were excluded because these measurements reflects patient effort and does not provide information about neural expiratory timings. Among these patients, we used a randomly selected five minute tracing to estimate the occurrence of non-entrained RT. The time between each neural effort (i.e., effective, and ineffective efforts) was determined to calculate neural breathing frequency. By using our previous validated algorithm each breath was classified as time triggered mandatory, patient triggered, ineffective, double triggering or non-entrained RT [22]. In addition, breaths were manually annotated as breath after a mandatory time triggered breath and before and after non-entrained RT breath. For each patient the percentage of RT breaths was calculated.

### Statistical analysis

The Shapiro-Wilk test was used to test for normal distribution. Normally distributed continuous data are presented as mean and SD. When the assumption of normality was not met, data are presented as median and 25–75 interquartile range (IQR). Categorical data are presented as percentage (%) of total. When comparisons between groups were made, continuous data were analyzed using the Mann-Whitney U test. Spearman’s rank correlation coefficient was used to calculate the correlation between two variables. Statistical analysis was performed with statistics software (IBM SPSS Statistics 27, IBM, Armonk, USA). *P* values below 0.05 were considered statistically significant.

## Results

In total 102 measurements (3110 min) from 67 patients (38 boys and 29 girls) were analyzed. Median age was 4.9 [1.8 ; 19,1] months and median weight 6.0 [4.7 ; 10.0] kg. Median duration of mechanical ventilation (MV) was 4.9 [3.7 ; 7.3] days, and median length of PICU stay was 5.9 [4.7 ; 9.0] days. NMB was used in 42 (63%) patients for a median duration of 36.0 [22.0 ; 52.4] hours. Fifty-four (81%) patients were admitted with primary respiratory failure, 7 (10%) after cardiac surgery, two (3%) for septic shock, and five (6%) patients were admitted for non-pulmonary reasons. Cuffed ETTs were used in 52 (78%) patients.

**Fig. 3:**
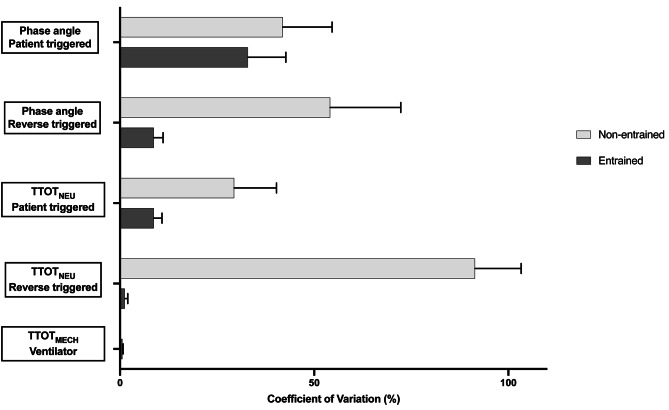
Variance of time triggered mandatory, patient triggered and reverse triggered breaths. Coefficient of variation for the breath interval and phase angles for ventilator breaths (TOT_MECH_), patient breaths (TOT_NEU_) and reverse triggered breaths (TOT_NEU_). Two bars are displaying the differences between entrained and non-entrained breaths. Coefficient of variation < 15% is considered entrainment. The highest coefficient of variation is seen in non-entrained reverse triggered breaths (91%)

In 12 recordings (12%) from 11 patients (16%) entrained RT was identified. During these 12 recordings 52 periods of entrained RT were observed. In thirty-nine recordings (38%) from 30 patients non-entrained RT was seen. Baseline demographics and ventilator settings for both study groups are shown in Table 2. TTOT_NEU_ CoV for entrained RT breaths 1.2% [0.6 ; 2.0] was lower compared to non-entrained RT breaths 91.4% [72.4 ; 103.3] (*p* < .001). Overall TTOT_NEU_ CoV (i.e. patient triggered and RT breaths combined) was lower for patients with entrained RT (4.3% [1.7 ; 10.8] compared to patients with non-entrained RT 29.4% [19.9 ; 40.3] (*p* < .001) (Fig. 3).

**Table 2:** Baseline demographics and ventilator setting entrained and non-entrained reverse triggering

	Entrained reverse triggering cohort	Non-entrained reverse triggering cohort	*p*
Baseline demographics			
N	11	30	
Recordings	12	63	
Age (months)	8.4 [2.5 ; 20.3]	4.9 [1.8 ; 19.1]	0.733
Weight (kg)	8.8 [5.1 ; 12.9]	6.0 [4.8 ; 10.0]	.386
Duration of MV (days)	3.2 [1.5 ; 4.9]	4.9 [3.8 ; 6.9]	.026
Length of PICU stay (days)	4.1 [2.5 ; 7.3]	5.9 [5.1 ; 7.9]	.020
Duration of NMB (hours)	4.4 [2.5 ; 18.5]	39.5 [24.3 ; 60]	< .001
Comfort B score	12 [10 ; 14]	14 [12 ; 18]	0.1
Ventilator settings			
Peak Inspiratory Pressure (cm H_2_O)	20 [18 ; 26]	20 [15 ; 23]	0.261
Positive End Expiratory Pressure (cm H_2_O)	5 [5 ; 6]	5 [4 ; 6]	0.566
Set Frequency (/min)	30 [25 ; 34]	25 [20 ; 30]	0.250
Inspiratory Time (sec)	0.65 [0.55 ; 0.90]	0.55 [0.55 ; 0.65]	0.034
Mode			
Pressure A/C	11	60	
Pressure SIMV	-	1	
Pressure Regulated Volume Control	-	2	
Time Cycled Pressure Limited A/C	1	-	

Data are presented as median (interquartile range)

MV = mechanical ventilation; PICU = paediatric intensive care unit; NMB = neuromuscular blockade; A/C = assist control; SIMV = synchronized intermittent mandatory ventilation

Patients with non-entrained RT had significantly more often prior use of NMB and for a longer duration of time (39.5 h [24.3 ; 60] vs. 4.4 h [2.5 ; 18.5]) (*p <* .001). Duration of MV (3.2 days [1.5 ; 4.9] vs. 5.9 [5.1 ; 7.9]) (*p* = .026) and length of PICU stay (4.1 days [2.5 ; 7.3] vs. 5.9 days [5.1 ; 7.9]) (*p* = .02) was significantly shorter among patients with entrained RT events compared with non-entrained RT.

### Entrainment and entrained reverse triggering

In all but one patient (11/12, 92%) we observed that the most common type of entrained RT was 1:1 ratio. Median TTOT_MECH_ CoV from time triggered mandatory breaths was 0.6% [0.2 ; 0.8], median TTOT_NEU_ CoV from entrained RT was 1.2% [0.6 ; 2.0] and from patient triggered breaths 8.7% [4.3 ; 10.8]. Median TTOT_NEU_ CoV from overall breaths was 4.3% [1.7 ; 10.8] (Fig. 3). Median phase angle for entrained RT was 110° [65 ; 184] and for patient triggered breaths 45° [29 ; 71] (*p* < .001). Median phase angle CoV from entrained RT was 8.7% [6.1 ; 11.1], median phase angle CoV from patient triggered breaths was 32.9% [18.1 ; 42.7] and from overall breaths (i.e. patient triggered and entrained reverse) 36.6% [22.2 ; 55.5] (Fig. 3). Median percentage differences between neural breathing rate differs and set mandatory breath rate was 4.5% [0.8 ; 11.4]. There was a significant relationship between TTOT_NEU_ CoV and decreasing difference between neural breathing rate and set mandatory breath rate (*r* = .89, *p* < .001). The Vte from time triggered mandatory breaths was significantly higher (7.8 ml/kg [7.5 ; 8.5] than during patient triggered breaths (6.7 ml/kg [5.4 ; 7.3]) and entrained RT breaths (6.6 ml/kg [5.9 ; 7.1] (*p* < .001). We did not observe breath stacking during entrained RT.

**Fig. 4:**
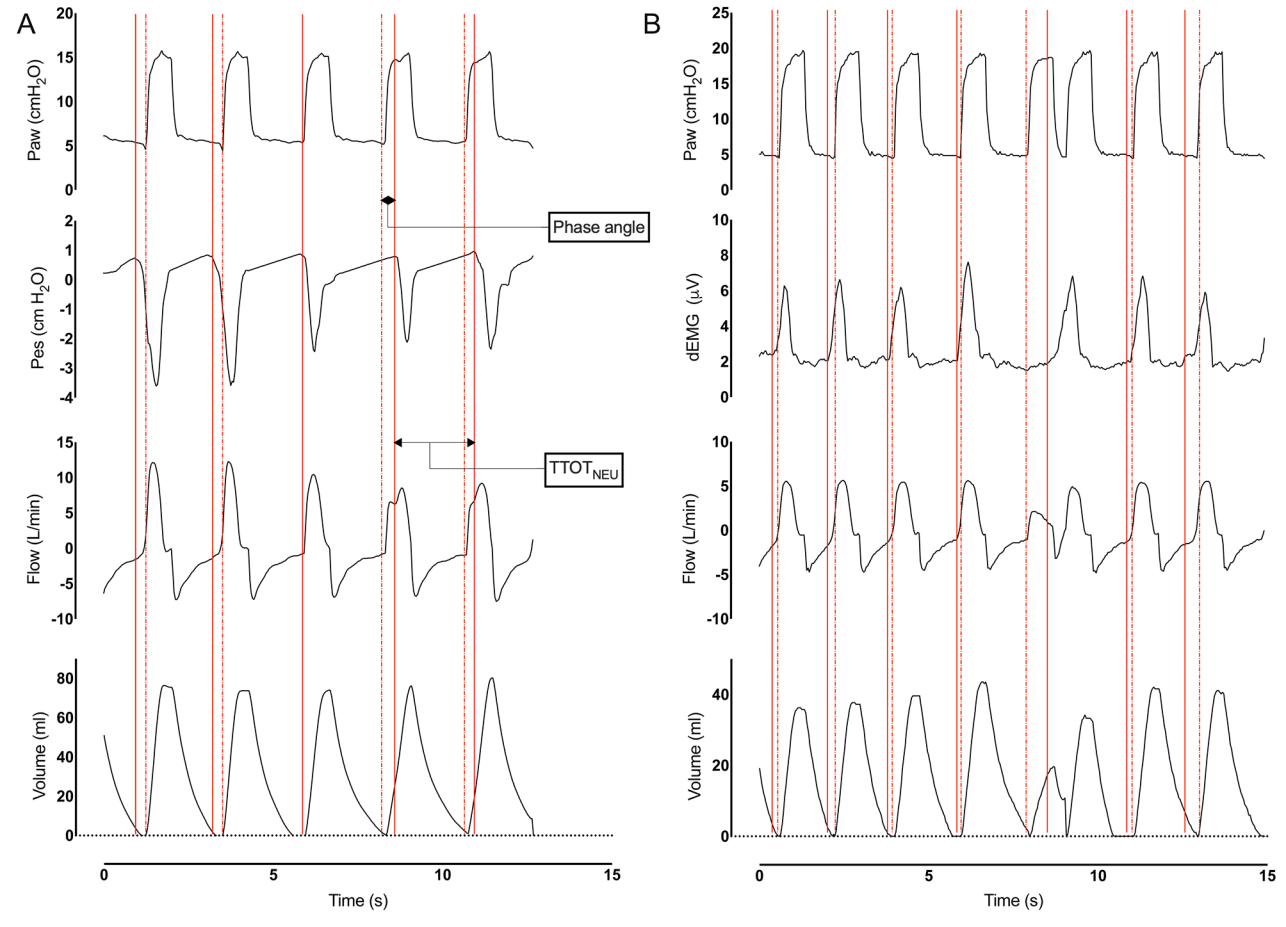
Representative examples of entrained and non-entrained reverse triggering. Examples of entrained and non-entrained reverse triggering. Phase angle is used to calculate the difference between the start of a patient of effort and ventilator pressurization. The breathing interval (start patient effort until a secondary patient effort) is expressed as TTOT_NEU_. Subject **A** is showing entrained reverse triggering. During respiratory entrainment the patient is cycling between patient triggered breaths and reverse triggered breaths. The first two breaths are patient triggered and the last 2 breaths are reverse triggered. For illustrative reasons only the first 2 out of 4 entrained reverse breaths are being displayed. Despite changing phase angles (positive to negative) the breathing interval remains constant. As shown; no excessive oesophageal pressure swings or breath stacking occurs. Patient **B** is showing non-entrained reverse triggering. Variance in phase angle and TOT_NEU_ are > 15%. During the non-entrained reverse triggering event two ventilator breaths are delivered. Despite the double triggered breaths tidal volume are smaller compared to tidal volumes during patient triggered ventilation. Breath stacking resulted in an increase of 20% of delivered tidal volume

In six patients entrained RT could be detected using oesophageal pressure tracings; this allowed us to calculate the pressure time product (PTP). Median PTP for an entrained RT breath was 0.5 cm H_2_O*s [0.4 ; 1.2] and for a single patient triggered breath 0.8 cm H_2_O*s [0.4 ; 1.1] (*p* = .12). Δ P_pes_ for a single entrained RT breath (2.9 cm H_2_O [2.3 ; 7.4]) was significantly smaller than the Δ P_pes_ for a single patient triggered breath (4.3 cm H_2_O [3.1 ; 5.5]) (*p* = .019)(Fig. 4). In the remaining 6 recordings (60%) entrained RT could be detected using dEMG. Median ΔdEMG for a single entrained RT breath was 2.5 µV [1.2 ; 4.2] and for a single patient triggered breath 1.8 µV [1.3 ; 2.4] (*p* < .001). The dEMG_INT_ for a single entrained RT breath was significant higher (0.8 µV*s [0.2 ; 1.6]) than a single patient triggered breath (0.4 µV*s [0.2 ; 0.5]) (*p* = .002).

### Non-entrained reverse triggering

Sixty-three measurements from 30 patients (13,767) breaths were available to determine the prevalence of non-entrained RT. We identified non-entrained RT in 62% of measurements, yielding a median percentage of non-entrained RT breaths of 1.1% [0.0 ; 4.5]. Median TTOT_NEU_ CoV from non-entrained RT was 91.4% [72.4 ; 103.3] and from patient triggered breaths 29.4% [19.9 ; 40.3] (Fig. 3). Median phase angle for non-entrained RT was 83° [25 ; 126] and for patient triggered breaths 101° [75 ; 131] (*p* < .001). Median phase angle CoV from non-entrained RT was 54.1% [36.7 ; 72.3], median phase angle CoV from patient triggered breaths was 41.9% [29.8 ; 54.6] (Fig. 3). Similar to patients in whom entrainment was identified, we observed a significant relationship between TTOT_NEU_ and decreasing difference between neural breathing rate and set mandatory rate (*r* = .73, *p* < .001).

After a breathing cycle with no breathing effort (i.e., time triggered mandatory breath) patients were 4.4 times more likely to develop a non-entrained RT event in the consecutive breathing cycle (*p* < .001). Neural expiratory time was significantly increased following the delivery of a time triggered mandatory breath (0.69 s [0.36 ; 1.05]) compared to patient triggered ventilation (0.63 s [0.39 ; 0.94]) (*p* = .042). In addition, baseline dEMG activity, ΔdEMG and dEMG_INT_ after a time triggered mandatory breath were all significantly lower than after patient triggered ventilation breathing (*p* = .008, *p* < .001, *p* < .001). We observed a significant relationship between the percentage of non-entrained RT and the difference between neural breathing rate and set mandatory rate (*r* = .71; *p* < .001). There was no significant correlation between set mandatory rate itself, ventilator set pressures (i.e., inspiratory pressure and PEEP) and percentage of non-entrained RT.

During analysis of patients dEMG-recordings, 380 double triggering (3.5%) events were observed. 47% of these double triggering events were patient triggered. In the remaining 53% double triggering was time triggered with the second breath being a patient effort (i.e., non-entrained RT). These non-entrained double triggering events showed no sign of respiratory entrainment (Fig. 3). Median Vte for double triggering was 12.1 ml/kg [7.4 ; 14.7] (Fig. 4). There was no difference between Vte for patient triggered or non-entrained reverse related double triggering (*p* = .967).

## Discussion

To our best knowledge, this is the first study reporting that RT in mechanically ventilated children caused by respiratory entrainment did not lead to breath-stacking, excessive transpulmonary pressure swings or increased pressure-time product. We also found that non-entrained RT occurred when the neural expiratory time was significantly increased after a time triggered mandatory breath. Double-triggering with breath-stacking occurred in non-entrained RT and not in entrained RT. Our data show that the clinical phenotype of RT is diverse and underscore the importance of identying non-entrained RT to reduce the potential injurious effects of this type of asynchrony.

Respiratory entrainment has been proposed as an important mechanism in the development of RT [1, 23]. In this type of neuromechanical coupling, a fixed ratio develops between an external stimulus (i.e., insufflation of the lung) and the patient respiratory rhythm with a decrease in breathing variabilty [6, 7]. These findings are similar to ours. Patients with entrained RT showed breathing variance, in both spontaneous and RT breaths, < 15% and close to the variance of the set mandatory frequency variance. Investigations in adults have demonstrated that entrainment in a 1:1 ratio could be established when the mandatory breath rate was set in range of the subjects own breathing frequency [6, 7]. Similar findings were made in neonates where respiratory entrainment was observed if the neural breathing frequency matched the set mandatory rate [24, 25]. These findings are compatible with our observations that the lowest breathing interval CoV, as an indicator for respiratory entrainment, was seen when the patient neural breathing frequency was matching set mandatory rate and within an offset of 1–11%. Because of the nature of our study we could not determine if changing the mandatory frequency could interrupt or induce RT. The mechanisms underlying respiratory entrainment are incompletely understood. Slowly adapting stretch receptors (SARs) responsible for preventing lung overinflation (i.e., the Hering-Breuer reflex) may contribute to the development of respiratory entrainment [8]. However, other reflexes are probably also involved as respiratory entrainment has also been described in patients post-lung transplantation [8]. In these patients, the feedback loop through the phrenic nerve is interrupted. We have also observed entrained RT in one post-lung transplant patient in our cohort. It may be surmised that the patterns of RT in our cohort may not be the result of respiratory entrainment, but merely delayed triggering. However during delayed triggering patients will have breathing variance comparable to a spontaneoeus breathing pattern (i.e., CoV > 15%). Patients with entrained RT showed a breathing variance < 15%, making delayed triggering less likely.

We also identified respiratory entrainment in the group with non-entrained RT. Patients with this clinical phenotype were less likely to experience single non-entrained RT if the set mandatory rate matched the patient’s own neural respiratory rate. In addition, our data showed that the CoV was the highest for non-entrained RT breaths, thereby confirming that there was no relation with respiratory entrainment. This suggest that other factors, alongside respiratory entrainment are responsible for single non-entrained RT. It has been proposed by some that single non-entrained RT represents premature triggering, as a complete expression of patient-ventilator asynchrony or as desynchronization [10, 11]. We postulate that other mechanisms may also contribute as the Hering-Breuer reflex also display physiological effects other than respiratory entrainment including prolongation up to 35% of the neural expiratory time following mechanical inflation of the lung through a mandatory breath [6, 7, 26]. We observed that patients were more likely to develop a single non-entrained RT event when the preceding breath was a mandatory breath. This means that when there is a prolonged neural expiratory time, the ventilator already initiates the new time triggered mandatory breath before the patient’s neural inspiration begins. While such an event may be interpreted as RT, it actually reflects disturbed neuromechanical coupling due to the mandatory breath. This can thus partially explain the occurrence of a single non-entrained RT event without respiratory entrainment. An alternative explanation could be the Head’s paradoxical reflex. Mediated by rapidly adapting pulmonary stretch receptors (RARs), an inspiratory effort is generated after lung insufflation instead of an expiration [27, 28]. During this reflex, augmented breaths are meant to prevent lung collapse and facilitating the first breath in neonates [27, 29–32]. Obviously, our study warrants further investigations into the underlying mechanisms.

Whether (entrained) RT contributes to ventilator induced lung injury (VILI) remains subject of debate. In our study population we observed a longer duration of MV, length of PICU stay and increased use of NMB in patients with non-entrained RT. Due to the design of this study we could not identify if these findings are related to non-entrained breaths or are merely an indicator of disease severity. Reverse triggering can cause breath-stacking leading to injurious volume delivery and excessive transpulmonary pressure swings contributing to regional lung stress [3, 12, 15]. We could not study transpulmonary pressure swings since we did not have oesophageal tracings in this part of the cohort. We did find that in non-entrained RT double triggering resulted in the delivery of large Vt, similar to double cycling resulting from premature termination. However, this study was not designed to determine if these large Vt contributed to lung damage. We did not observe breath-stacking during entrained RT. At the same time, it may also be surmised that entrained RT itself facilitates patient-ventilator synchrony and that increased expiratory diaphragmatic activity may prevent atelectasis and lung collapse [33].

Our data show that the clinical phenotype of RT is diverse, making it important to identify non-entrained RT to reduce the potential injurious effects of breath-stacking, caused by this type of asynchrony. This can be achieved calculating the single breath phase angle, breath cycle interval and CoV. RT as a part of respiratory entrainment should have little variance in breathing interval CoV for both spontaneous patient triggered breaths as for entrained RT breaths. The variance of the phase angle during neuromechanical coupling should be higher comparing to the variance in breathing interval, because during entrainment patient triggered breaths and RT breaths are occurring in an alternating pattern. During non-entrained RT there is no neuromechanical coupling and hence no relation with respiratory entrainment. Under these circumstances variance in breathing interval CoV should be higher then 15% [9]. This assumption is supported by previous work and our data [1]. Thus, phase angle calculations and breathing interval CoV may aid in discriminating between entrained RT and non-entrained RT.

There are some limitations to our study that need to be addressed. First, our data represents a single-center study which included a relative small sample size of 67 patients, potentially limiting generalizability. Second, because our study is a secondary phyiology analysis of prospective collected data it was not designed to determine the positive or negative effects of respiratory entrainment and RT on clinical outcome. In addition, the effect of age on respiratoy feedback mechanisms are not clear. Having a relative young study population may have influenced our findings limiting extrapolation of our findings to older children and adults. Fourth, patients with non-entrained reverse triggering received NMBs for a longer duration. However by not including a total severity of illness it is not clear if non-entrained reverse triggering is a direct consequence of NMBAs or a marker of disease severity. Fifth, by not having oesophageal manometry in the second cohort we could not study transpulmonary pressure swings and the potential additional lung stress. And lastly we made 30 min recordings and because patient-ventilator interaction is variable during the course of mechanical ventilaton we may have over- or underestimating the true prevalence of reverse triggering.

## Conclusions

Respiratory entrainment causes reverse triggering in mechanically ventilated children without breath stacking or excessive pleural pressure swings. We could not relate respiratory entrainment to non-entrained reverse triggering breaths. Our data support the concept that reverse triggering is not one entity but a clinical spectrum with different mechanisms and consequences.

## Data Availability

No datasets were generated or analysed during the current study.

## Abbreviations

CoV: Coefficient of variation
CSV: Continuous spontaneous ventilation
ΔdEMG: Amplitude EAdi signal
dEMG: Transcutaneous recording of the electromyographic diaphragm signal
dEMG_INT_: Integrated EAdi signal
ΔPes: delta oesophageal pressure
EAdi: Electrical activity of the diaphragm
ETT: Endotracheal tube
IRB: Institutional Review Board
IQR: Interquartile range
NMB: Neuromuscular blockade
MV: Mechanical ventilation
PAP: Pressure above PEEP
PEEP: Positive end expiratory pressure
Pmean: Mean airway pressure
PS: Pressure support
PTP: Esophageal pressure-time product
PVA: Patient-ventilator asynchrony
RARs: Rapidly adapting pulmonary stretch receptors
RT: Reverse triggering
SARs: Slow adapting stretch receptors
SD: Standard deviation
TTOT_NEU_: Time interval (sec) breathing cycle patient triggered breath
TTOT_MECH_: Time interval (sec) breathing cycle time triggered mandatory breath
VILI: Ventilator induced lung injury
VOXP: Ventilator open XML protocol
Vt: Tidal volume
Vte: Expiratory tidal volume
